# Isolated taste disorders in patients referred to a flavor clinic with taste and smell loss

**DOI:** 10.1002/brb3.2071

**Published:** 2021-02-16

**Authors:** Dovile Stankevice, Alexander Wieck Fjaeldstad, Therese Ovesen

**Affiliations:** ^1^ ENT Department Regional Hospital West Jutland Holstebro Denmark

**Keywords:** electrogustometry, etiology, olfaction, taste disorder

## Abstract

**Background and Aim:**

Approximately five percent of the general population are suffering from taste disorders. Usually, subjective loss of taste is caused by smell disorders; thus, isolated loss of the sense of taste is relatively rare. Despite the influence on quality of life, isolated taste disorders (ITD) are sparsely represented in literature and clinical research. In particular, there is need for sufficient diagnostic and treatment algorithms. Aim of study is to add further information to the sparse literature on ITD and suggest an appropriate diagnostic algorithm for ITD.

**Methods:**

We present a retrospective study on patients with ITD diagnosed at the Flavour clinic, ENT department, Regional Hospital West Jutland, between 2017 and 2020. All patients underwent a thorough rhinolaryngological and otoneurologic examination, including olfactory and gustatory assessment, and a wide spectrum of biochemical, microbiological, and radiological examinations.

**Results:**

In total, 522 patients referred due to smell and/or taste disorders, 423 (81%) complained of a subjective taste disorder, only 19 (3.4%) patients were diagnosed with ITD. According to etiology, the patients were categorized in following groups: medicine‐related (25%), mixed (21%), iatrogenic (21%), idiopathic (11%), radiotherapy‐related (11%), and autoimmune/inflammatory (11%). Based on etiology, individualized treatment was recommended with very discouraging results.

**Interpretation:**

Diagnostics of taste disorders is a delicate and expertise demanding task. The mechanisms underlying isolated loss of taste are heterogeneous. ENT and otoneurologic examination, and specific olfactory and gustatory testing are required in all patients, whereas biochemical, microbiological, and radiological examinations are only necessary on an individual basis.

## Graphical abstract

A study on isolated taste disorders, which is a complaint with multiple different potential medical etiologies. The mechanisms underlying isolated loss of taste are heterogeneous. We suggest diagnostic work‐up and possible treatment.

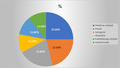

## 1 INTRODUCTION

Taste and smell are chemical senses tightly connected with quality of life. Loss of one or both of these senses often leads to significant reduction of mental and often physical well‐being (Hur et al., 2018; Kuba et al., 2018; Zabernigg et al., 2010). Approximately 15%–20% of the population suffers from smell disorders, which is often mistaken for a combined smell and taste loss (Fjaeldstad et al., 2018; Soter et al., 2008). Five percent of the general population complains of reduced taste function (hypogeusia) without contemporary smell problems—often an age‐related problem—whereas complete taste loss (ageusia) is rare (Fark et al., 2013). Compared to smell disorders, the scarcity of literature on isolated taste disorders (ITD) is remarkable.

According to Fark et al. (2013), the most common etiologies of ITD are idiopathic, posttraumatic, and postoperative. Other frequent etiologies are adverse effects related to various medications and ITD associated with medical causes and viral infections (Fark et al., 2013; Hunt et al., 2018; Rademacher et al., 2020; Schiffman, 2018). Additionally, ITD is often reported during cancer treatment with chemotherapy and/or radiotherapy of the head and neck. Chemotherapy causes disturbances of taste function in 69.9% of patients during treatment (Zabernigg et al., 2010). ITD related to radiotherapy is induced by massive taste bud loss (Deshpande et al., 2018). Late local complications after radiotherapy in the head and neck region such as xerostomia, dysphagia, caries, and oral candidiasis can also have a negative impact on taste function (Deshpande et al., 2018). Furthermore, specific deficiencies of vitamin B1, B6, B12, zinc, as well as anemia, and kidney failure are also assumed to cause taste dysfunction (Malaty & Malaty, 2013).

Taste disorders often have a negative impact on food intake and nutrition status besides reducing compliance of medication and quality of life. Association between depression and taste disorders has been described among 19% of patients. An even higher prevalence of taste disorders (23.7%) was found among patients with major depression (Hur et al., 2018).

As only few clinics offer systematic work‐up, little consensus exists on ideal diagnostical algorithms for ITD. Often the taste disturbances are merely represented in subjective questionnaires without accompanying objective or psychometric measures. In light of the potential of reversing some of the mechanisms underlying ITD such as cessation/substitution of medication, amplification of food tastants, and Zinc or vitamin supplements, it is surprising how little attention is paid to these conditions (Fark et al., 2013; Schiffman, 2018).

It is of major importance to diagnose taste disorders and to develop diagnostic as well as treatment algorithms. In this context, the rarity of ITD is challenging. Therefore, the aims of the present retrospective study were as follows: 1. Add further information to the sparse literature on ITD, and 2. Suggest an appropriate diagnostical algorithm for ITD.

## 2 SUBJECT AND METHODS

The Flavour Clinic at the ENT department, Regional Hospital West Jutland, Denmark, was established in 2016 as the only nationwide center for smell and taste disorders. The clinic receives patients referred from practicing ENT specialists. Baseline work‐up before referral consists of CT of the nose and sinuses, allergy screening, and endoscopic evaluation of the nose and pharynx.

At the first visit, all patients filled in a standard questionnaire about age, gender, variety of symptoms, smoking habits, medical history, and medication, as well as the Sino‐nasal outcome Test (SNOT‐22), and Major Depression Inventory (MDI). Mini‐Mental State Examination (MMSE) was performed by a trained nurse.

All patients underwent a thorough ear–nose and throat examination, including endoscopy of the nose, as well as flexible pharyngo‐laryngo‐endoscopy, and otoneurologic examination. Swabs from the tongue were administered to diagnose a possible Candida infection. Olfactory patency was assessed using "Sniffin’ Sticks" extended battery, consisting of a TDI score (threshold, discrimination, identification) to identify patients with isolated taste disorders. Olfactory loss was assessed by comparing TDI scores to an age‐matched normative population.

The first patients with isolated taste disorder (ITD) were initially tested with Filter Paper Test (FPT), but after developing a new method to test taste function with higher reproducibility, the Taste Drop Test (TDT) was introduced to the diagnostic algorithm (Fjaeldstad et al., 2018). Here, a list of options consisting of "neutral," "sweet," "sour," "salty," and "bitter" is presented to the patient, who has to decide on one of these taste qualities (forced multiple choice). The patient has to rinse the mouth with neutral water after each presentation of tastants. Four tastants are included in the test: sweet (sucrose), salty (NaCl), sour (citric acid), and bitter (quinine). Each tastant is presented in 10 levels of dilution. A drop with each of four different tastant is presented on the tongue in the lowest concentration (dilution step 10). After correctly identification of a tastant, the one step lower concentration is presented to the patient in the subsequent round. If this lower concentration is not recognized, the previously correctly identified tastant concentration is repeated in the subsequent round. When two succeeding presentations of a given tastant concentration are correctly identified without correct identification of the lower concentration, a tastant sensitivity score is registered for that tastant. The correct identified tastant is replaced with a neutral stimulation (water) in the subsequent semi‐randomized round until tastant sensitivity scores for all four tastants are registered (Fjaeldstad et al., 2018). Ageusia was defined as a score < 18, and hypogeusia as score < 25 using TDT (Fjaeldstad et al., 2018). In case of FPT, the corresponding scores for ageusia and hypogeusia were below nine and below 17 (hypogeusia), respectively (Fark et al., 2013).

In our cohort, taste drops were presented bilaterally, except in patients suspected of postoperative taste disorder due to nerve lesions (e.g. unilateral or bilateral glossopharyngeal nerve lesion after tonsillectomy). Such cases were tested meticulously in order to reveal a possible side of nerve lesion. The tongue was divided in four quadrants: right anterior (2/3), left anterior (2/3), right posterior (1/3), and left posterior (1/3) according to the innervation of the tongue of the facial and the glossopharyngeal nerves, respectively. Posteriorly, we applied FPT separately on the right and the left side. Anteriorly, patients were tested with TDT on the right and the left side. To further diagnose possible nerve lesions, patients underwent electrogustometry (Sensonics International) applied to similar anatomic tongue locations as described above. Electrogustometric measurements were categorized as pathologic in the following cases: the difference of the thresholds between the left and the right side exceeded 4 dB; or the threshold value was abnormal: glossopharyngeal nerve > 14 dB and chorda tympani > 8 dB (Tomita & Ikeda, 2002; Tomita et al., 1986).

Biochemical screening included: hemoglobin, ferritin, transferrin, TSH, Hb A1c, Zn, Cu, Fe, creatinine, sodium, potassium, vitamin B1, B6, and B 12 levels to identify underlying anemia, endocrine disorders, and vitamin or mineral deficiencies.

MRI of the cerebrum (1.5 T) was only performed after individual clinical assessment and in most cases due to unclear etiology of ageusia. Some patients had imaging performed before referral from, for example, neurologists.

### 2.1 Ethical approval

Data were collected after regional approval from the Danish Data Protection Agency. The retrospective design did not require ethics approval (Danish Committee Act, Section 14, Subsection 2).

## 3 RESULTS

Of the first 522 consecutive patients seen at the Flavour clinic, 19 were diagnosed with ITD (3.4%). In comparison, 330 patients were diagnosed with isolated smell disorder and 87 had a combined taste and smell disorder. Patients often had complained of both smell loss (*n* = 496) and taste loss (*n* = 423). Demographics of the 19 patients with ITD are listed in Table 1.

**TABLE 1:** Demographics of patients with ITD

*N* = 19	
Age	Mean: 56 years (32–80 years)
Gender	5 (26%) male – 14 (74%) female
Duration of symptoms	
≤1‐year	6(32%)
1 ≤ 2 years	6(32%)
2 to ≤5 years	5(26%)
˃5 years	2(10%)
Comorbidity	
None	2
Cardiovascular	7
Metabolic	7
Depression	3
Autoimmune	2
Cancer	2
Neurological	1
Anemia	2
Actual medication	
None	3
Antihypertensives + statins	3
Antihypertensives + statins + Metformin	2
Antihypertensives + statins + antidepressants	1
Antidepressants	2
Methotrexate	1
Statins	1
Antihypertensives	1
Others	5

Of the ITD patients, five were men (26%) and 14 women (74%); mean age of 56 years ranging from 32 to 80 years. The duration of symptoms varied from 6 months to 8 years. Two thirds had symptoms of taste disorders for 6–24 months before they first presented at the clinic. Furthermore, the majority suffered from various comorbidity, and only three patients were without medication.

The results of the taste tests are listed in Table 2.

**TABLE 2:** Results of specific taste tests

	FPT simple	TDT simple	Advanced taste tests			
			FPT Posterior: left/right	TDT Anterior: left/right	Electrogustometry Posterior: left/right	Electrogustometry Anterior: left/right
*N* (%)	6(42)	9(47)	4(21)	—	—	—
Min	2	12	0/0	18/20	18/6 dB	−1/6.5 dB
Max	12	25	8/8	26/24	>34/>34 dB	>34/>34 dB
Median	6	19	5/5	21.5/22.5	8.75/19 dB	24.5/27.5 dB

In total, nine patients showed ageusia (47%), and ten were diagnosed as hypogeusia (53%). Ageusia was more often diagnosed using FPT (55.6%) than TDT (44.4%), and one patient was diagnosed with hypogeusia using FPT.

Lateralized TDT and electrogustometry were applied to diagnose the site of lesion in four suspected iatrogenic cases. Three patients suffered from taste loss after tonsillectomy, two of them were diagnosed with bilateral glossopharyngeal nerve injury. Additionally, in both cases decreased unilateral chorda tympani sensitivity was found. The last patient showed signs of unilateral chorda tympani and glossopharyngeal nerve affection. Here, we found decreased asymmetric electrogustometry thresholds only on the left side, both anteriorly and posteriorly. This did not correlate with the TDT and FPT results, which were higher on the opposite side of the tongue. These discrepancies may be due to the patient's disseminated sclerosis.

All TDI scores were normal, and all patients had normal cognitive function according to MMSE (Table 3). However, some had various sino‐nasal complaints according to SNOT 22. Swabs from the tongue revealed Candida species in three patients of whom only one had objective signs of infection. Systemic antimycotic treatment did not improve the taste function in any of the three patients.

**TABLE 3:** Results of MMSE, SNOT 22, MDI, TDI, microbiology, and blood tests

	MM SE	MDI	SNOT 22	TDI	Candida swab	Biochemistry										
						Hg	Transferin	Ferritin	TSH	Hb Ac1	Vit. B1	Vit. B6	Vit. B 12	Fe	Cu	Zn
*N*	19	19	19	19	16	13	9	10	11	9	7	8	12	4	6	11
%	100	100	100	100	84	68	47	53	58	47	37	42	63	21	32	58
Min	26	0	4	29	—	—	—	—	—	—	—	—	—	—	—	—
Max	30	55	49	37.5	—	—	—	—	—	—	—	—	—	—	—	—
Median	29	6	17	32.5	—	—	—	—	—	—	—	—	—	—	—	—
Neg.	—	—	—	—	13	—	—	—	—	—	—	—.	—	—	—	—
Pos.	—	—	—	—	3		—	—	—	—	—	—	—	—	—	—
Normal	—	—	—	—	—	11	8	8	11	6	6	4	10	3	3	9
High	—	—	—	—	—	2	1	1	0	1	1	3	1	0	3	0
Low	—	—	—	—	—	0	0	1	0	2	0	1	1	1	0	2

Blood tests showed low Zinc concentrations in two cases; one patient had low vitamin B6, and one had vitamin B12 deficiency (Table 3). Zinc supplement, 20 mg per day, was without effect. Vitamin B6 and B12 deficiencies were treated with vitamin B complex for several months, but did not improve the sense of taste.

Eight patients went through 1.5 T MRI of the cerebrum. Three demonstrated pathology: one patient had signs of vestibular neuritis, which was without relation to the taste disturbance. Another patient with known multiple sclerosis had supratentorial lesions. In the last case, sequelae in the frontoparietal area were found due to previous surgical removal of a meningioma. The brain stem was normal in all cases.

**FIGURE 1:**
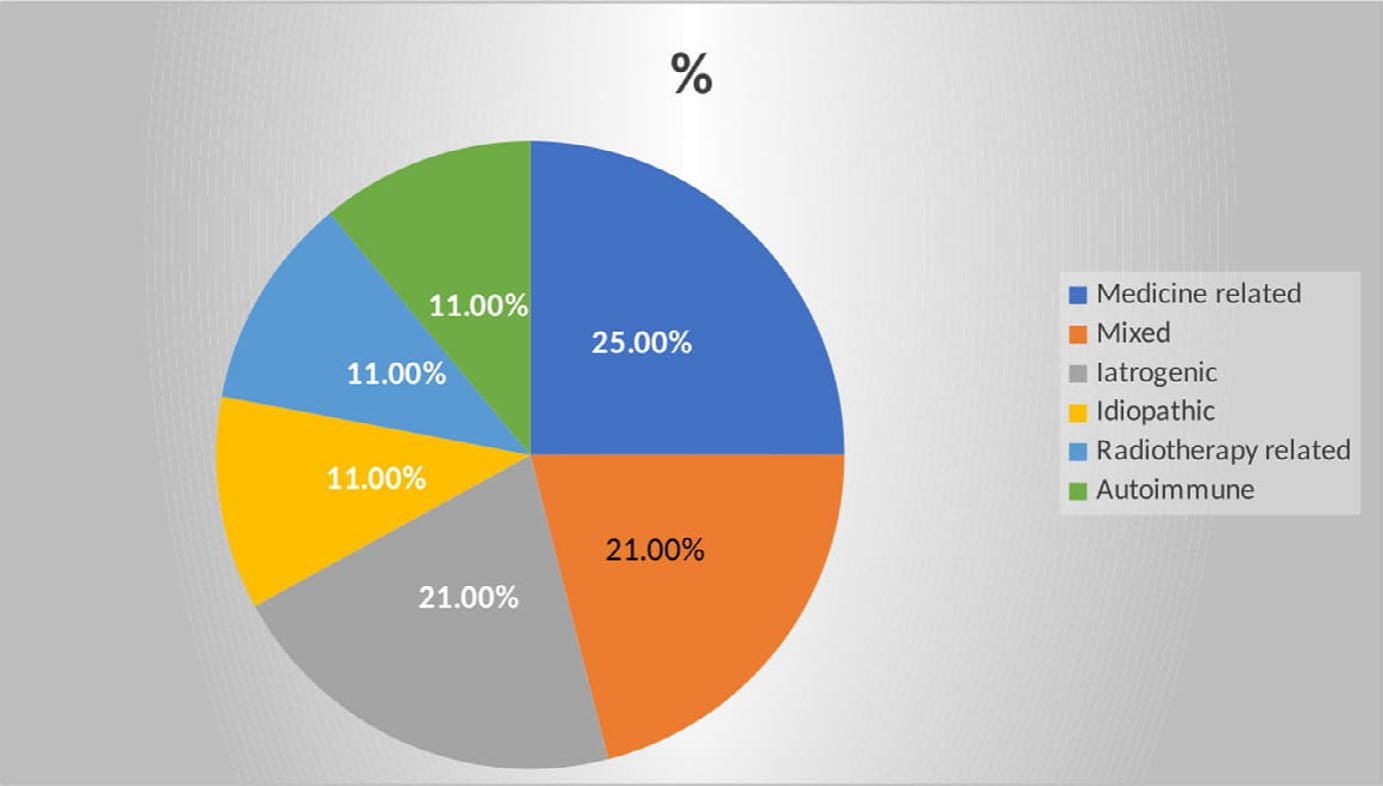
Etiology of ITD

Based on patient history and the work‐up program, the most probable causes/mechanisms underlying ITD in this cohort could be categorized in six main groups: mixed (more than one cause), idiopathic, medicine‐related, iatrogenic, radiotherapy‐related, and autoimmune/inflammatory (Figure 1). None of the patients had lost taste sensation following a head trauma. In four cases (21%), we identified more than one potentially competing etiological factor (mixed). Current medication was suspected to be involved in all four patients with mixed etiology. Two cases were classified as idiopathic. Of the remaining 13 patients, five were medicine‐related (25%), four cases (21%) were iatrogenic, that is, related to surgery, two cases were related to radiotherapy, and two patients suffered from autoimmune diseases.

Of the four mixed ITD cases, two patients suffered from type 2 diabetes and received medication reported to be associated with taste disturbances (metformin, statins, antihypertensives). The third patient was treated with chemotherapy 1 year prior to taste loss in relation to a viral infection. This patient was also treated with statins. The last case lost her sense of taste along with vestibular neuritis—she was also treated with antihypertensives (Rademacher et al., 2020; Schiffman, 2018).

Medication was considered to be the only cause of taste loss in five cases besides three of the possible causes in the four mixed cases, that is, a total of eight patients. The following medications were involved: metformin, ramipril, losartan, corodil, amlodipine, atorvastatin, simvastatin, and terbinafine which are known to induce taste disturbances (Danish Pharmaceutical Information Ltd., 2020; Rademacher et al., 2020; Schiffman, 2018). The sense of taste was not affected immediately after initiation of the medication, but after months/years. The three patients treated with antidepressants had other time‐related causes of the loss of taste (radiotherapy, surgery). Pausing of the medication for 3 months did not improve the sense of taste.

Two patients lost the sense of taste in relation to radiotherapy due to tonsillar and tongue carcinomas, respectively. Both patients complained of xerostomia.

In two cases, the taste disturbances were considered to of autoimmune origin: rheumatoid arthritis; furthermore, one of the patients had previously had Steven–Johnson's syndrome as complication to treatment with Methotrexate.

Four patients were diagnosed with **iatrogenic** taste loss: Three cases were reported after tonsillectomy and one patient reported tastes loss after directly laryngoscopy. Three of the patients were complaining about xerostomia, and one had burning mouth syndrome.

## 4 DISCUSSION

In the present study, we found that out of 522 consecutive patients with subjective smell and taste disorders, only 19 (3.4%) were diagnosed with ITD. The typical ITD patient was middle aged, female, suffering from various comorbidity and receiving commonly used medication. Adverse or side effects of medication were suspected to be involved in a total of eight cases (42%), followed by iatrogenic induced ITD in 21%. Specific treatment according to biochemical and microbiological findings as well as pausing medication for 3 months did not improve of the sense of smell in any of the patients.

In other retrospective studies, the rarity of ITD was also reported (Fark et al., 2013; Hunt et al., 2018). Thus, Fark et al. (2013) and Hunt et al. (2018) found 185/4,680 (4%) and 7/358 (2%), respectively, with ITD in their clinics. It means, literature on ITD is restricted to case series like this current study. With regard to demography, our results are in line with previous studies.

In our cohort, a heterogeneous range of comorbidities was observed: autoimmune, for example, oral lichen planus and rheumatic arthritis; endocrine, for example, diabetes mellitus; neurological, for example, disseminated sclerosis; cardiovascular disease, for example, arterial hypertension and hypercholesterolemia. In a study with 3,204 participants (428 diabetics, 2,776 controls), diabetics had higher prevalence of smell impairment compared to controls, whereas taste disorders were not observed (Rasmussen et al., 2018). However, only screening of the sense of taste with saline and kinin was applied. In contrast, a recent study focusing on diabetes mellitus type 2 and taste disorders demonstrated generally decreased taste function among patients, who was not affected of diabetic complications and did not take any medication which could affect smell or taste including antidiabetics. Furthermore, no correlations were found between decreased taste sensation and gender, duration of disease, and glycemic control, while a relationship between decreased taste and age was noticed (Pugnaloni et al., 2020).

Autoimmune diseases such as rheumatoid arthritis and Sjӧgren's syndrome are usually associated with complains such as xerostomia, burning mouth syndrome, halitosis, and poor dental status. Singh et al. (2019) demonstrated a 50% prevalence of taste disorders, xerostomia, and burning mouth syndrome among Sjӧgren's patients compared to non‐Sjögren controls; 15% and 26% of the Sjӧgren's patients had ageusia or hypogeusia, respectively, whereas taste disorders were found in 10% of non‐Sjӧgren's patients. Furthermore, recent data comparing patients suffering from rheumatoid arthritis with healthy controls demonstrated decreased olfactory and gustatory function independent of disease activity and duration of rheumatoid arthritis (Walliczek‐Dworschak et al., 2020).

In contrast to our data, other studies have reported idiopathic cases as the most prevalent cause, that is, between one third, and a half of all ITD patients (Fark et al., 2013; Hunt et al., 2018). There may be several reasons for this discrepancy: First, some authors used self‐reported taste disorders without objective examination or basic work‐up such us subjective screening tools, full‐mouth test, taste strips. This may distort statements about underlying etiologies. The same accounts for patient history as many patients are referred to specialized smell and taste clinics after several years, and potential links to infections or toxicity may be forgotten. Even with a thorough clinical work‐up, it can be difficult to appoint the exact causes of ITD among the patients because of longstanding symptoms.

The growing polypharmacy particularly in elderly patients induces a high risk of side effects. The single drug versus drug–drug interactions is easier to detect due to numerous national and international medical drug interactions databases. Concomitant drugs can alter each other's bioavailability and pharmacological effects (Schiffman, 2018). Seven (37%) patients from our study patients used the same combination of atorvastatin and amlodipine. According to the Danish national medical drug database (medstad.dk), about 11% of the Danish population are using statins (Danish Health Data Protection Agency, 2020). A total of 8 out of 19 patients (42%) used medication known to have potential of inducing taste disturbances, such as terbinafine, metformin, ACE inhibitors, statins (atorvastatin), calcium channels blockers (amlodipine), and propofol. As the underlying biochemical mechanisms are still poorly understand, no standard treatment for drug‐induced chemosensory disorders exists except from identification, and cessation or pausing medications (Du et al., 2018; Schiffman, 2018). We found no positive effect on taste function of a 3 months pause (if medically sound) of relevant medication including statins (atorvastatin) and metformin. For comparison, we have not been able to identify studies of pausing medication on taste function in the literature.

Despite numerous surgical procedures with potential of neural damage‐inducing ITD, only few studies have focused on ITD as a postoperative complication. Surgical damage of the glossopharyngeal nerve during tonsillectomy, and the chorda tympani during middle ear surgery resulting in high rates of short‐lasting taste impairment is well known, but rarely reported. Fortunately, the majority of such complaints disappears within 3–6 months after surgery. Permanent and long‐lasting symptoms of parageusia and dysgeusia after tonsillectomy occur in approximately one percent of cases (Heiser et al., 2010, 2012) and in approximately 17% of cases after cochlear implantation (Mikkelsen et al., 2017).

Caries, xerostomia, dysphagia, oral candidiasis, and taste disorders are frequently reported during radiotherapy in the head and neck region depending on the local affection of taste buds as well as damage of salivary glands and xerostomia (Deshpande et al., 2018; Epstein et al., 2016). Taste disorders associated with radiotherapy typically develop after 4 weeks of treatment, and the majority recovers after 6–12 months after completed radiotherapy (Deshpande et al., 2018). Unfortunately, some patients never fully regain normal taste function. Salty and bitter tastes are the most affected, whereas sweet taste seems to be the most resistant and the least affected (Epstein et al., 2016). Higher doses of radiation against the tongue cause higher rates of taste disorders (Epstein et al., 2016; Nguyen et al., 2012). Furthermore, patients treated with radiotherapy alone experience a greater taste loss compared with chemotherapy or combined radiotherapy/chemotherapy (Zabernigg et al., 2010). Proton therapy is associated with lower risk of acute toxicities. Minimal damage of taste buds on the tongue and preserved salivary glands should dramatically reduce complains of IDT during radiotherapy (Deshpande et al., 2018).

Additionally, several studies reported a strong association between chemotherapy regimens and taste disorders during chemotherapy. About 70%–75% patients during chemotherapy were affected by taste disorder. Severity of taste disorder differed depending on used chemotherapy treatment paradigms (Kuba et al., 2018; Zabernigg et al., 2010).

Considering treatment of ITD, our findings are very discouraging. We experienced that pausing medication did not improve the sense of taste. Antimycotic treatment in case of positive Candida test was also without significance. Finally, despite corrected dosages of oral Zinc and B vitamins, respectively, treatment results were unsuccessful, which is in contrast to a previous randomized clinical trial (Heckmann et al., 2005). This calls for future studies of the specific underlying molecular biological processes in the taste buds in order to find more specific treatments.

## 5 CONCLUSION

In this study, we presented work‐up and findings among patients with ITD. ITD was diagnosed in 3.4% of patients complaining of taste and smell loss referred to a specialized flavor clinic. As such, isolated taste disorders were rare in comparison with isolated smell disorders and combined taste and smell disorders. Comparison between studies is impeded by relatively small populations and very different work‐up. We recommend that patients with taste complaints are examined early after onset of symptoms and that the work‐up includes thorough medical history, relevant objective/psychometric testing of the gustatory function not relying entirely on patient‐reported data. Additional analysis should be individualized as microbiology, biochemistry, and imaging in general does not add any information. In general, ITD can be categorized as a peripheral sensory disorder involving either the taste buds or afferent neurons.

Taste disorders often have severe negative effects on quality of life and nutrition, why knowledge of diagnostics and treatment is important. Our attempts to improve the sense of taste were very discouraging, and future studies unravelling molecular biological mechanisms in the taste buds are needed in order to identify new treatment strategies.

## CONFLICT OF INTEREST

The authors declare they have no conflict of interest nor financial support for the study. The second author has received research salary funding from Arla Foods (Viby, Denmark) and the Central Region Denmark. The sponsors had no say, roles, or responsibilities in relation to the study, including (but not limited to) the study design, data collection, management, and analysis.

## AUTHOR CONTRIBUTION

DS, AWF, and TO had the idea for the study. AWF was responsible for the data base setup. DS examined the patients and conducted the clinical follow‐up. DS wrote the initial draft for the manuscript with input from AWF and TO. DS, AWF, and TO reviewed the manuscript.

### Peer Review

The peer review history for this article is available at https://publons.com/publon/10.1002/brb3.2071.

## Data Availability

The data are not publicly available due to restrictions in the approval by the Danish Data Protection Agency. Data that support the findings of this study are available on request from the corresponding author with certain limitations due to the GDPR requirements for privacy.
